# Supplementary material for: Updating an allocentric goal from lateralised egocentric visual memories
Source: Nat Commun. 2026 Mar 6;17:3594. doi: 10.1038/s41467-025-67545-3 (PMC13096645; doi:10.1038/s41467-025-67545-3)
Supplement: Supplementary file 1 — Supplementary information [file 41467_2025_67545_MOESM1_ESM.pdf]

Supplementary Figure 1 (part 1)

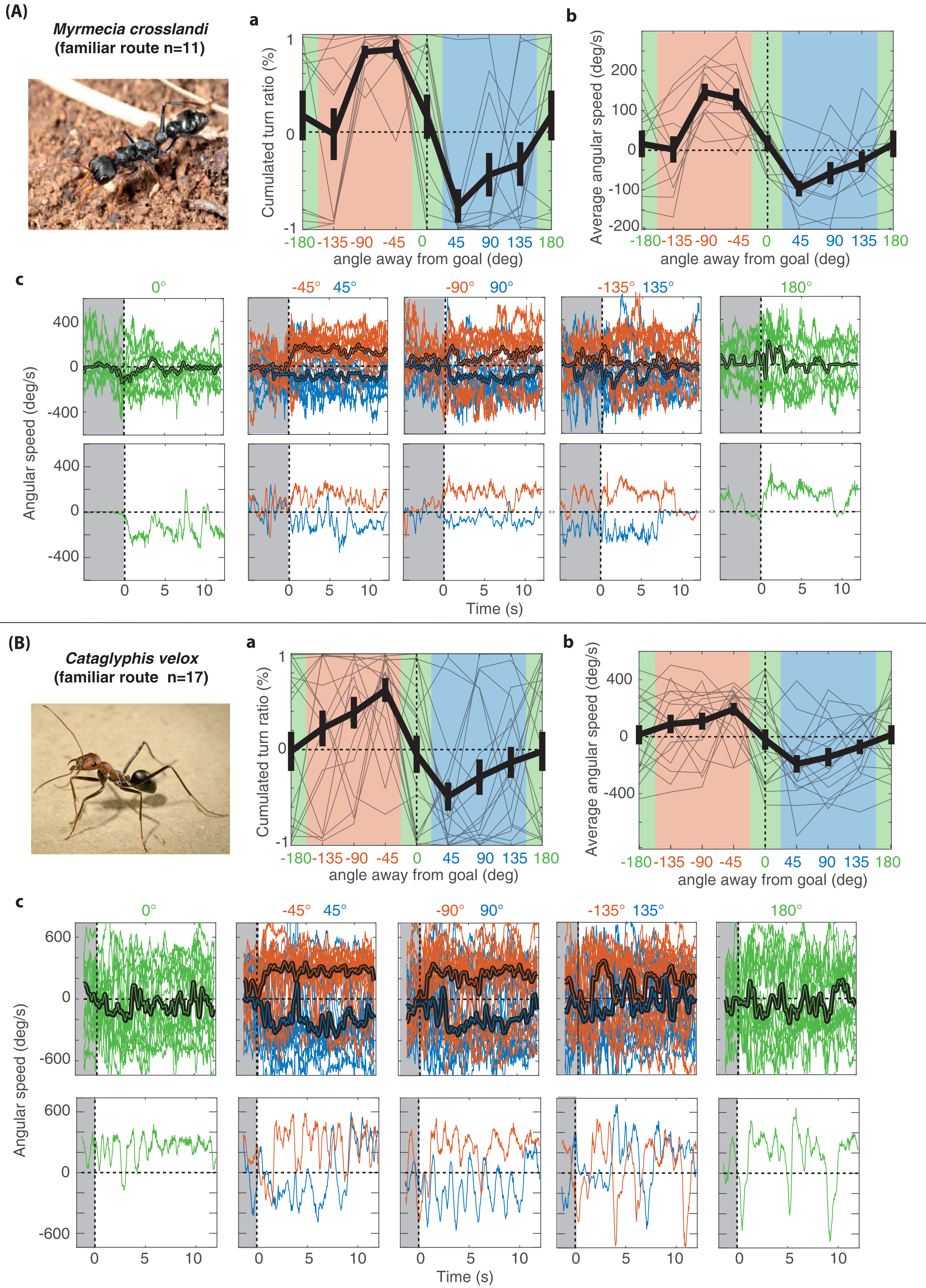

Supplementary Figure 1 (part 2)

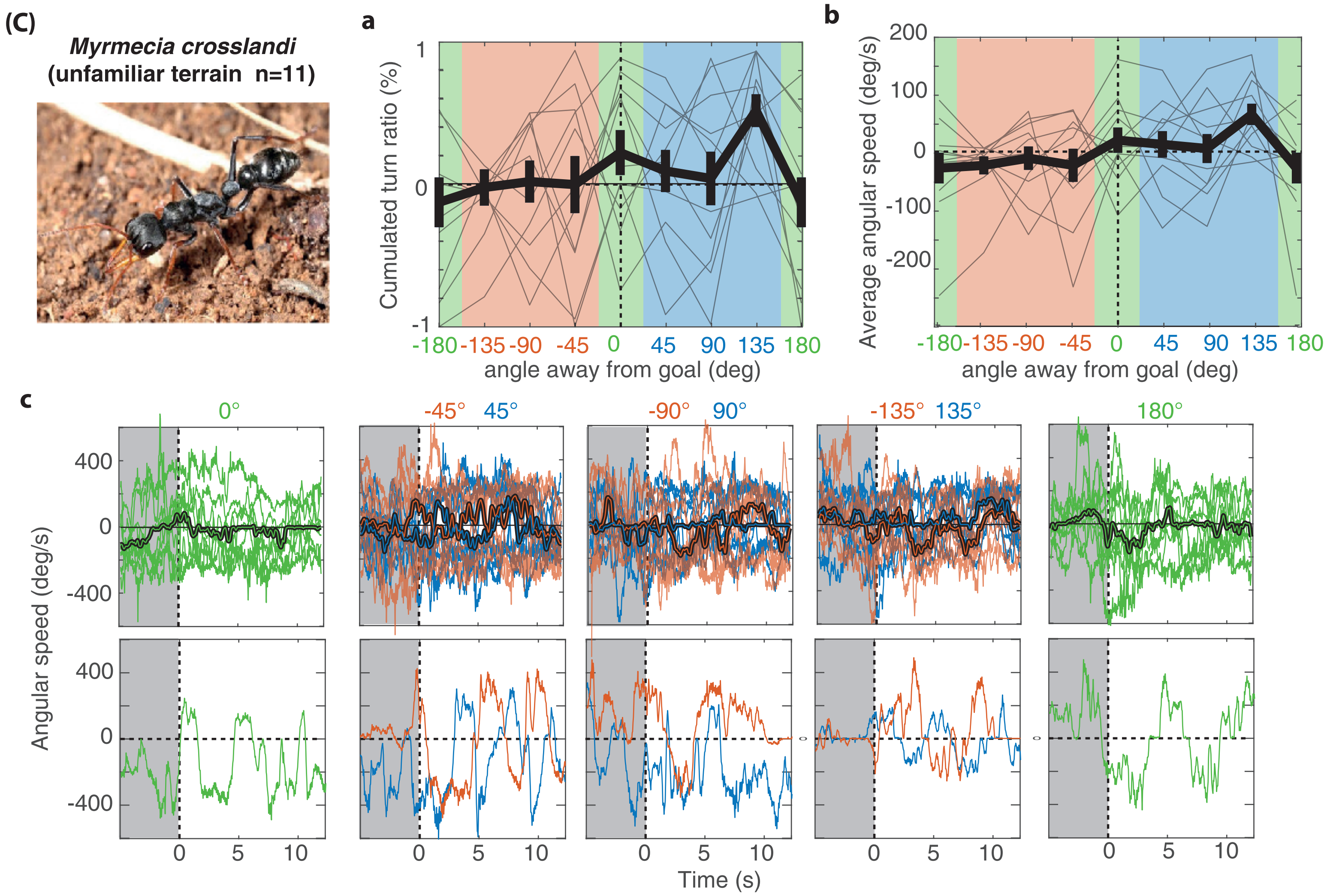

Supplementary Figure 1. Additional Data for Fig. 1

Supplementary data from the experiment shown in Fig. 1, for Myrmecia croslandi (A) and Cataglyphis velox (B) on familiar terrain, and Myrmecia croslandi on unfamiliar terrain (C). Homing ants were captured at the nest and fixed on the trackball on their familiar route in 8 different compass orientations (see Fig.1a). Red and blue colors indicate that the correct route direction lies to the right and left, respectively. Green correspond to the route (0°) and anti-route (180°) directions.

a, b. Turn ratio ((right – left) / (right + left)), with right/left angles derived from the integral of absolute angular velocity) (a) and mean angular speed (b) across the eight compass orientations. Thin lines represent individual data averaged over 12 seconds of recording; thick lines indicate the population average (± s.e.m.). Positive and negative values indicate right and left turns, respectively.

c. Angular speed over time for the eight compass orientations. The grey area marks the period before the bucket was lifted, when the ant could not yet see the surrounding scene. Top row: individual traces (thin lines) and population average (thick line). Bottom row: a single example individual.

Supplementary Figure 2

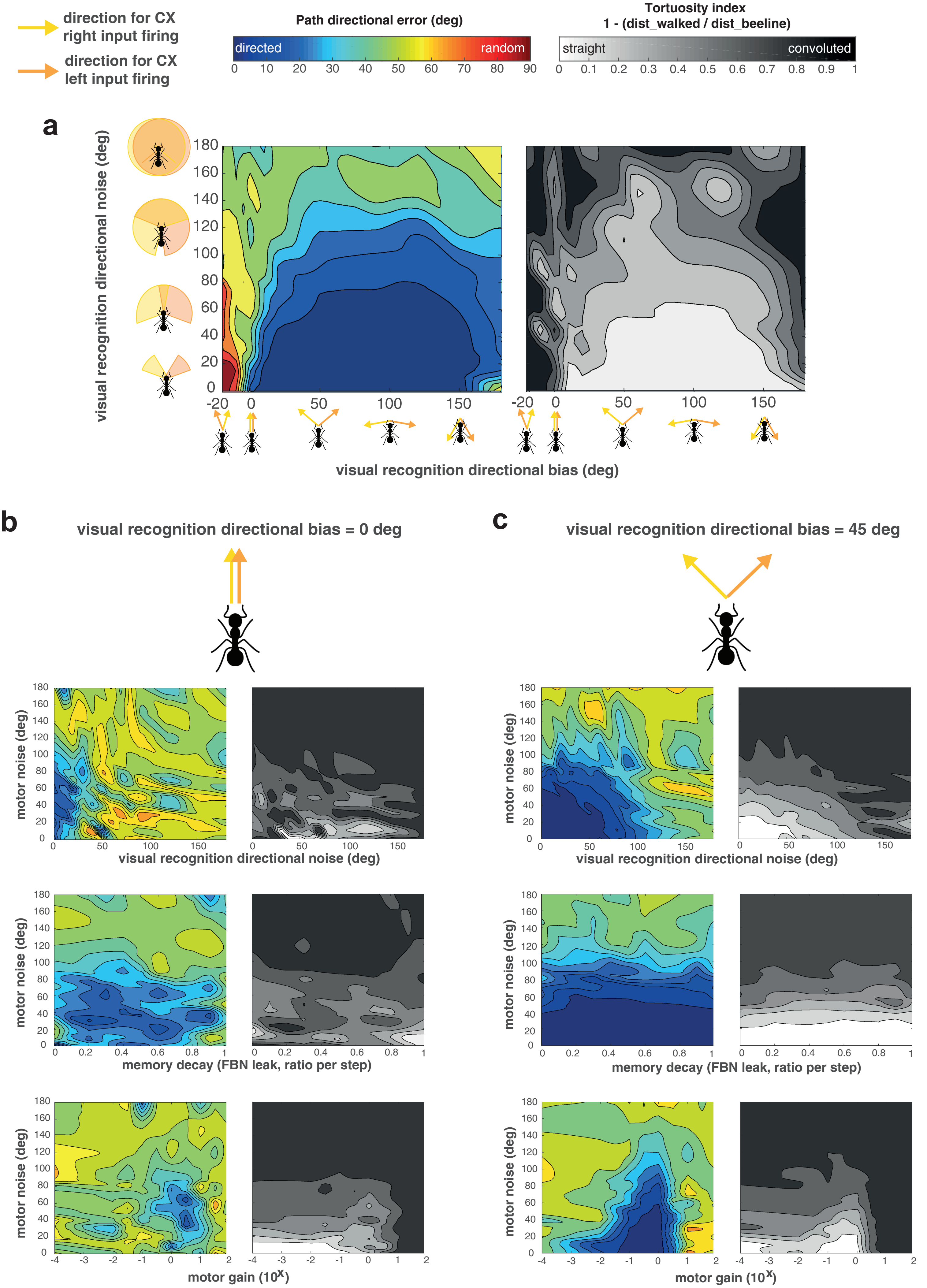

Supplementary Figure 2. Parameter exploration of the Central Complex model (see Fig. 3).

This shows a parameter exploration for the Central complex (CX) model presented in **Fig. 3** (see **Supplementary Figure 2** for details of the circuitry). - Path directional error (absolute angular error between start-to-arrival and start-to-goal directions) and path tortuosity (index = *1 - (beeline_distance/distance_walked)*) after 200 steps are shown according to various parameter ranges. For each point on the map, all the other parameters are chosen to maximise for lowest path directionality error.

a. Same as **Fig. 3d**, except that, here, the search explores the entire parameter space to find regimes minimizing path directional error. In **Fig. 3d**, unrepresented parameters (motor noise, motor gain, memory decay) were fixed at average values, which led to routes in the opposite direction (i.e., directional error ~180°) when directional bias < 0. In contrast, the full parameter searches here can select combinations (typically with high motor noise) that produce very tortuous paths—resulting in random directions (directional error ~90°) – rather than routes in opposite directions (~180°). This shows that when visual familiarity bias is < 0, no parameter regime can yield straight, goal-oriented paths (directional error ~0°). Note that straight, goal-oriented paths emerge as long as the visual familiarity direction bias is > 0, that is, if the left hemisphere inputs correlate with moments when the nest is on the left, and vice versa.

b. Visual familiarity directional bias is fixed at a value of 0°, meaning that both CX inputs respond maximally when the agent is facing the goal direction. Note that in this condition, regions of low path directional errors (blue) and region of low path tortuosity (white) do not overlap. This means that one cannot obtain straight, goal-directed paths if left and right CX inputs respond when the nest is located in front.

c. Visual familiarity directional bias is fixed at a value of +45°, meaning that left and right CX inputs respond maximally when the agent is oriented 45° to the right or left from the goal direction, respectively. Note that regions with low path directional errors (blue) and regions of low path tortuosity (white) overlap well, showing a very large range of parameters for which we can obtain straight, goal-directed paths. We found the robustness to parameters remarkable: the model copes with motor noise up to 80°, visual familiarity direction noise up to 90°, is insensitive to its vector-memory decay and operates across several orders of magnitude for the gain.

Parameters' description:

Visual familiarity directional bias: Indicates the absolute angle away from the goal at which visual familiarity signals (i.e., the CX inputs) are highest, assuming 0° indicates the correct goal direction. 0° indicates that both left and right inputs fire when the nest direction is aligned with the current body orientation. Inversely, 180° indicates that left and right input fire when the nest is right behind. Positive values (between 0° and 180°) indicate that the left and right inputs fire when the nest direction is on the left and right hand side respectively (the extent of the angular bias is given by the value). Negative values (between 0° and -180°) indicate a reversal, so that left and right input fire when the nest direction is on the right and left hand side respectively. ***Visual familiarity directional noise:*** Represents the extent of a systematic deviation from the visual familiarity directional bias angle. It is implemented by shifting the input curve response (horizontal arrows in **Fig. 3b**) around its mean (given by the 'directional bias') at each time step by random values drawn from a normal distribution with standard deviation given by 'directional noise'. It can be seen as representing a directional noise when storing visual memories. High directional noise means that the input signal will occasionally respond strongest when oriented in the other direction than indicated by the visual familiarity directional bias. Robustness to visual familiarity directional noise indicates that the orientation of the body does not need to be precisely controlled during memory acquisition. ***Motor noise*****:** at each time step, a directional 'noise angle' is drawn randomly from a Gaussian distribution of *± SD = motor noise*, and added to the agent's current direction. ***Memory decay*****:** proportion of Fan-shaped Body Neurons (FBN, see Supplementary Figure 2 for details) activity lost at each time step: For each FBN: $\text{Activity}_{(t+1)} = \text{Activity}_{(t)} \times (1 - \textit{memory decay})$. This corresponds to the speed at which the memory of the vector representation in the FBN decays. A memory decay = 1 means that the vector representation in the FBN is used only for the current time step and entirely overridden by the next inputs. A memory decay = 0 means that the vectors representation acts as a perfect accumulator across the whole paths (as in Path Integration), which is probably unrealistic. ***Motor gain***: Sets the gain to convert the motor neuron signals (see Supplementary Figure 2 for details) into an actual turn amplitude (*turn amplitude = turning neuron signal × gain*). Note that here, the motor gain is presented across orders of magnitude. One order of magnitude higher means that the agent will be one order of magnitude more sensitive to the turning signal.

Supplementary Figure 3

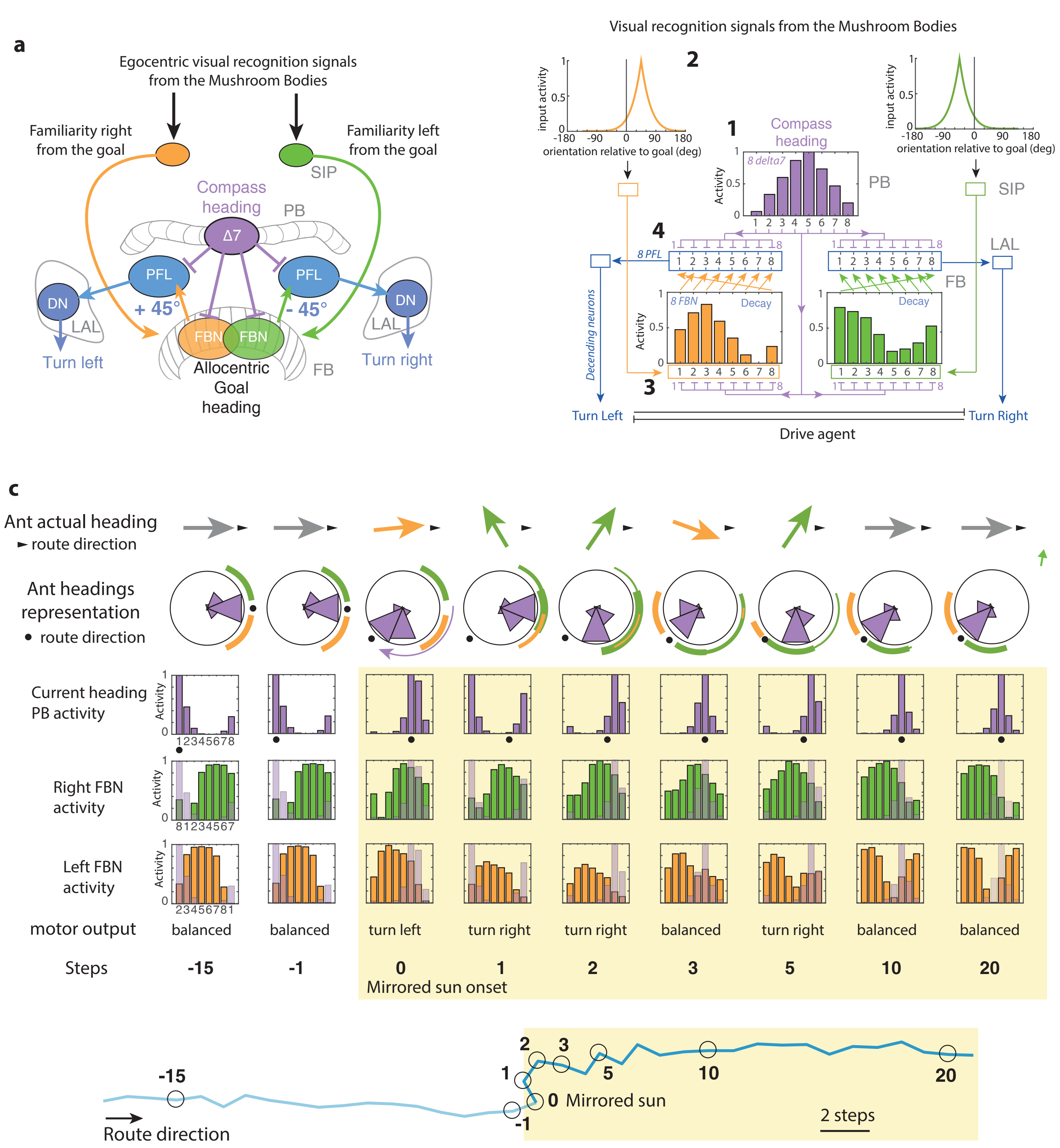

Supplementary Figure 3. Details of the CX model's circuitry (bee path integration version).

a-b. General scheme of the Central Complex (CX) (**a**) and corresponding detailed circuitry (**b**). This model exploits the same basic circuit as the CX model used for path integration in bees [1,2], except that FB input indicate visual familiarity rather than speed of movement.

b1. Current heading direction is modelled in the Protocerebral Bridge (PB) as a bump of activity across 8 delta7 (Δ7) neurons forming a ring-attractor (purple), as observed in insects [1,3,4]. Each neuron responds maximally for a preferred compass direction, spaced 45° apart from the neighbour neurons (neuron 1 and 8 are functionally neighbours, closing the ring structure). Change in the agent's current compass orientation results in a shift of the bump of activity across the 8 neurons (we did not model how this is achieved from sensory cues, see [5–7] for studies dedicated on this.

b2. Visual familiarity signals fire according to the agent orientation relative to the goal direction. Here the input curve indicates that right and left signals fire maximally when the agent is oriented 50° (in average) left and right from its goal respectively (but see Fig. 3 and Supplementary Figure 1 for variation of these parameters: 'directional bias' and 'directional noise').

b3. These lateralised input signals excite two dedicated sets of Fan-shaped body neurons (FBN). These FBNs are simultaneously inhibited by the current heading representation (purple), resulting in two negative imprints of the current heading activity across the FBNs, which can be viewed as two 'view-based vectors'. FBNs show some sustained activity so that, across time, successive imprints are superimposed, thus updating the 'view-based-vectors' (as for Path integration, except that this sustained activity is not crucial). The sustainability of such a 'view-based vector' depends on the FBN activity's decaying rate, which can be varied in our model and has little incidence on the agent's success (Supplementary Figure 1, parameter decay).

b4. Motor control is achieved using the same circuitry as for Path integration [1]. On each brain hemisphere, neurons (called PFL in some species), compare the current compass heading (purple) with their version of the FBN 'view-based-vector'. Crucially, both FBN representations are neurally shifted by 1 neuron (as if rotating the view-based-vector by 45° clockwise or counter-clockwise depending on the hemisphere), resulting in an overall activity in the PFL

(sum of the 8 PFL) indicating whether the view-based-vector points rather on the left- (higher resulting activity in the left hemisphere) or right-hand side (higher resulting activity in the right hemisphere). The PFL neurons sum their activity on descending motor neurons (DN), which difference in activity across hemispheres triggers a left or right turn of various amplitude, given a 'motor gain' that can be varied to make the agent more or less reactive (Supplementary Figure 1 for detailed parameter description). Numbers on the left of Δ7, FBN and PFL indicates neurons numbers. Letters on the right indicate brain areas (SIP: Superior Intermediate Protocerebrum, PB: Protocerebral Bridge, FB: Fan-shaped Body, LAL: Lateral Accessory Lobe).

c. Same as Fig. 4c, with added details of the PB (purple) and right and left FB (yellow and orange) neural activity (shown both as histograms and on circular plots). Note that the FBNs order has been shifted (2,3,4,5,6,7,8,1 and 8,1,2,3,4,5,6,7) and inhibition exerted by the PB is represented (overlaid transparent purple, 1,2,3,4,5,6,7,8) as happens in the left and right PFL1 neuron (**b4**). This way, the strength of the motor signal for turning right and left– which correspond to the sum of non-inhibited right and left PFL activity – can be inferred by looking at the area covered by non-occluded yellow and orange FBN columns respectively.

With manipulation such as rotating the current compass information, it becomes apparent that motor decision results from complex dynamics between two main factors: 1- how strong are the left and right visual input signal updating the view-based-vectors representation (represented by orange and yellow glow around the actual ant heading arrows), which depend on whether the agent is oriented left or right from its goal and 2- how well the current heading representation (PB) matches the rotated left and right shifted FB view-based-vector current representations

Supplementary Figure 4

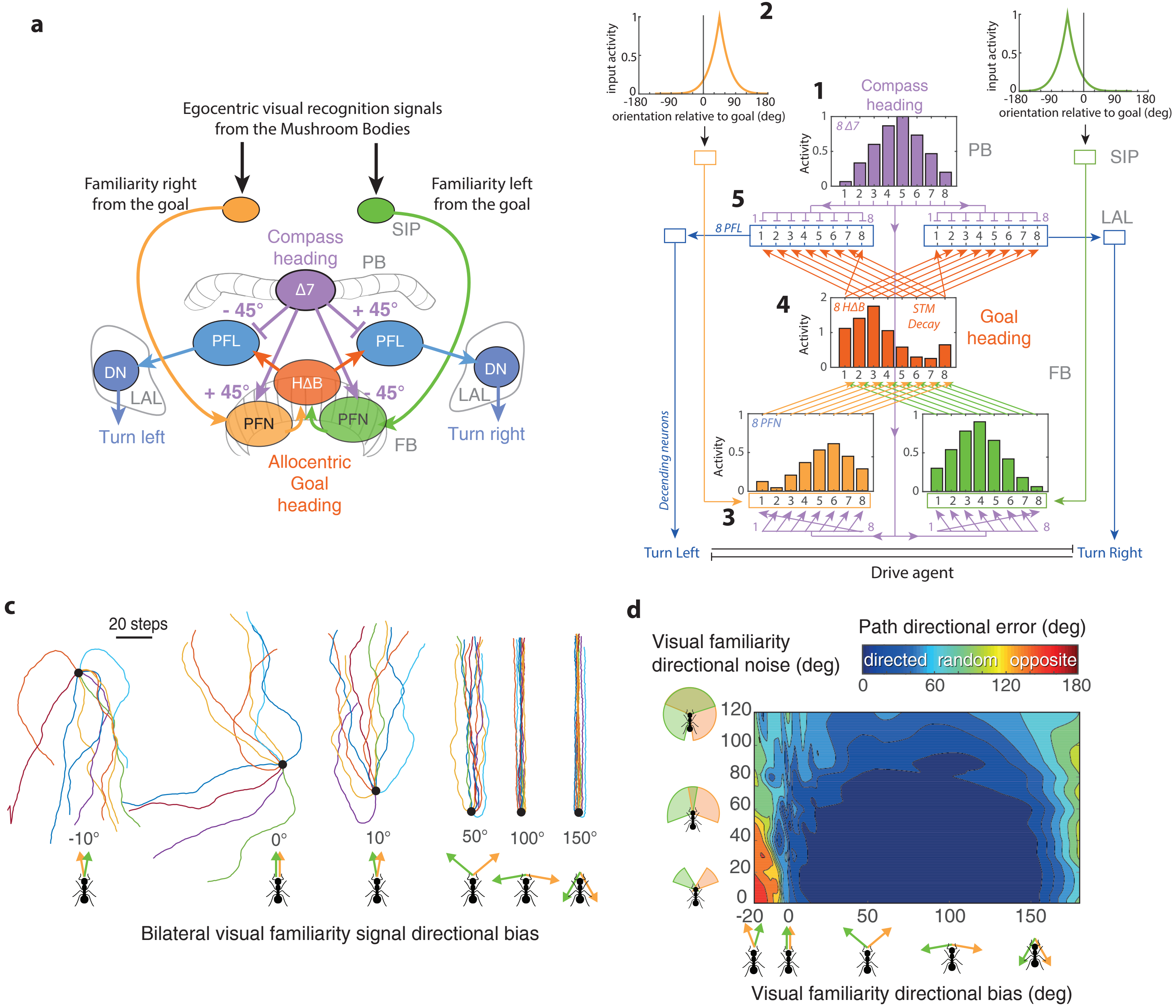

Supplementary Figure 4. Details of the CX model's circuitry (fly allocentric travel version).

a-b. General scheme of the Central complex (CX) (**a**) and corresponding detailed circuitry (**b**). This model exploits the same basic circuit as the CX model used for building an allocentric travel direction representation[8,9], except that FB input indicate visual familiarity rather than speed of movement.

b1. Current heading direction is modelled in the Protocerebral Bridge (PB) as a bump of activity across 8 delta7 (Δ7) neurons forming a ring-attractor (purple), as observed in insects [3]. as the later are described Each neuron responds maximally for a preferred compass direction, 45° apart from the neighbour neurons (neuron 1 and 8 are functionally neighbours, closing the ring structure). Change in the agent's current compass orientation results in a shift of the bump of activity across the 8 neurons (we did not model how this is achieved from sensory cues, see [5–7] for studies dedicated on this.

b2. Visual familiarity signals fire according to the agent orientation relative to the goal direction. Here the input curve indicates that right and left signals fire maximally when the agent is oriented 50° (in average) left and right from its goal respectively (but see Fig. 3 and Supplementary Figure 1 for variation of these parameters: 'directional bias' and 'directional noise').

b3. These lateralised input signals excite two dedicated sets of Fan-shaped body neurons called PFNs. These PFNs populations are simultaneously gated by two ±45° shifted versions of the current heading representation (purple) – thanks to delta7 (Δ7) neurons excitatory proprieties[12] –, resulting in two imprints of the current heading activity shifted in opposite direction. The direction of the shift is set so that the PFNs population receiving left from the goal familiarity signal are shifted towards the right (and vice versa), that is, in the theoretical direction of the goal.

b4. Both PFNs population sum their activity in a single population of hDeltaB (HΔB) cells[8], forming a single representation of the goal heading, which can be viewed as a short term 'view-based vector'. HΔB cells shows sustained activity so that, across time, successive imprints are superimposed, thus updating the 'view-based-vector'. The sustainability of such a 'view-based vector' depends on the HΔB activity's decaying rate, which can be varied in our

model and has little incidence on the agent's success (Supplementary Figure 1, parameter decay).

b5. Motor control is achieved using the same circuitry as in bees [1] or flies[10,11]. On each brain hemisphere, PFL neurons, 'compare' the current compass heading (purple) – thanks to Δ7 neurons inhibitory proprieties[12] – with two ±45° shifted versions of the current goal heading (HΔB population), resulting in an overall activity in the PFL (sum of the 8 PFL) indicating whether the view-based-vector points rather on the left- (higher resulting activity in the left hemisphere) or right-hand side (higher resulting activity in the right hemisphere) of the current heading. The PFL neurons sum their activity on descending motor neurons (DN), for which difference in activity across hemispheres triggers a left or right turn of various amplitude, given a 'motor gain' that can be varied to make the agent more or less reactive (Supplementary Figure 1 for detailed parameter description). Numbers on the left indicate neurons numbers. Letters on the right indicate brain areas (SIP: Superior Intermediate Protocerebrum, PB: Protocerebral Bridge, FB: Fan-shaped Body, LAL: Lateral Accessory Lobe).

c, d. same as **Fig. 3 c,d**. Paths resulting given different directional biases (**c**). Path directional error (absolute angular error between start-to-arrival beeline, and start-to-goal direction) after 200 steps, as a function of the visual familiarity 'directional bias' (x axis) and 'directional noise' (y axis) (**d**). Straight route headings robustly emerge as long as left and right inputs send a signal when the body is oriented right and left from the goal, respectively (i.e., directional bias > 0°) but not if both inputs send a signal when facing the goal (i.e., directional bias = 0°). Orientation towards the opposite direction emerges if left and right inputs signal inversely, that is, when the body is oriented right and left from the goal respectively (i.e., directional bias < 0°). Robustness to visual familiarity directional noise indicate that the direction in which views are learnt does not need to be precisely controlled.

References

1. Stone, T. *et al.* An Anatomically Constrained Model for Path Integration in the Bee Brain. *Current Biology* **27**, 3069-3085.e11 (2017).
2. Le Moël, F., Stone, T., Lihoreau, M., Wystrach, A. & Webb, B. The Central Complex as a Potential Substrate for Vector Based Navigation. *Front. Psychol.* **10**, (2019).

3. Honkanen, A., Adden, A., Freitas, J. da S. & Heinze, S. The insect central complex and the neural basis of navigational strategies. *Journal of Experimental Biology* **222**, jeb188854 (2019).
4. Seelig, J. D. & Jayaraman, V. Neural dynamics for landmark orientation and angular path integration. *Nature* **521**, 186–191 (2015).
5. Green, J. & Maimon, G. Building a heading signal from anatomically defined neuron types in the Drosophila central complex. *Current Opinion in Neurobiology* **52**, 156–164 (2018).
6. Kim, S. S., Hermundstad, A. M., Romani, S., Abbott, L. F. & Jayaraman, V. Generation of stable heading representations in diverse visual scenes. *Nature* **576**, 126–131 (2019).
7. Pfeiffer, K. & Homberg, U. Organization and Functional Roles of the Central Complex in the Insect Brain. *Annual Review of Entomology* **59**, null (2014).
8. Lyu, C., Abbott, L. F. & Maimon, G. Building an allocentric travelling direction signal via vector computation. *Nature* **601**, 92–97 (2022).
9. Lu, J. *et al.* Transforming representations of movement from body- to world-centric space. *Nature* **601**, 98–104 (2022).
10. Mussells Pires, P., Zhang, L., Parache, V., Abbott, L. F. & Maimon, G. Converting an allocentric goal into an egocentric steering signal. *Nature* **626**, 808–818 (2024).
11. Westeinde, E. A. *et al.* Transforming a head direction signal into a goal-oriented steering command. *Nature* **626**, 819–826 (2024).
12. Franconville, R., Beron, C. & Jayaraman, V. Building a functional connectome of the Drosophila central complex. *eLife* **7**, e37017 (2018).

Supplementary Table 1. Statistics for Fig. 1c,d

Signrank test	Turn ratio, against 0		Turn ratio, against 0			Time ratio: On route > Unfamiliar
	Cataglyphis	On route	Myrmecia	On route	Unfamiliar	Myrmecia
Facing orientation	N	Pvalue	N	Pvalue	Pvalue	Pvalue
180	17	0.5189	11	0.4155	0.7676	0.0336
-135	17	0.0591	11	0.5845	0.7935	0.0014
-90	17	0.0102	11	0.0005	0.4492	0.0068
-45	17	0.0006	11	0.0010	0.5171	0.0269
0	17	0.9434	11	0.5195	0.3203	0.0508
45	17	0.0060	11	0.0049	0.7402	0.0005
90	17	0.0488	11	0.0415	0.5508	0.0161
135	17	0.2315	11	0.1602	0.9985	0.0615

*Grey shading indicates where no effect is expected
