# Supplementary material for: Updating an allocentric goal from lateralised egocentric visual memories
Source: Nat Commun. 2026 Mar 6;17:3594. doi: 10.1038/s41467-025-67545-3 (PMC13096645; doi:10.1038/s41467-025-67545-3)
Supplement: Supplementary file 2 — Reporting Summary [file 41467_2025_67545_MOESM2_ESM.pdf]

nature portfolio

Corresponding author(s): Antoine Wystrach

Last updated by author(s): Jun 11, 2025

Reporting Summary

Nature Portfolio wishes to improve the reproducibility of the work that we publish. This form provides structure for consistency and transparency in reporting. For further information on Nature Portfolio policies, see our Editorial Policies and the Editorial Policy Checklist.

Statistics

For all statistical analyses, confirm that the following items are present in the figure legend, table legend, main text, or Methods section.

n/a	Confirmed	
[ ]	[x]	The exact sample size (n) for each experimental group/condition, given as a discrete number and unit of measurement
[ ]	[x]	A statement on whether measurements were taken from distinct samples or whether the same sample was measured repeatedly
[ ]	[x]	The statistical test(s) used AND whether they are one- or two-sided<br>*Only common tests should be described solely by name; describe more complex techniques in the Methods section.*
[ ]	[x]	A description of all covariates tested
[ ]	[x]	A description of any assumptions or corrections, such as tests of normality and adjustment for multiple comparisons
[ ]	[x]	A full description of the statistical parameters including central tendency (e.g. means) or other basic estimates (e.g. regression coefficient) AND variation (e.g. standard deviation) or associated estimates of uncertainty (e.g. confidence intervals)
[ ]	[x]	For null hypothesis testing, the test statistic (e.g. F, t, r) with confidence intervals, effect sizes, degrees of freedom and P value noted<br>*Give P values as exact values whenever suitable.*
[x]	[ ]	For Bayesian analysis, information on the choice of priors and Markov chain Monte Carlo settings
[x]	[ ]	For hierarchical and complex designs, identification of the appropriate level for tests and full reporting of outcomes
[x]	[ ]	Estimates of effect sizes (e.g. Cohen's d, Pearson's r), indicating how they were calculated

Our web collection on statistics for biologists contains articles on many of the points above.

Software and code

Policy information about availability of computer code

Data collection	The data in this study was obtained using ant mounted on a custom trackball system. Raw data consists of the movements of the ants on the ball, which were obtained by two sensors quantifying the movment of the ball along the three axis.
Data analysis	Raw data of the movements of the ants across time were analysed using Matlab R2016b. We also performed computational modelling using Matalb. Raw data and Matlab codes are available on a Github repository. https://github.com/antnavteam/lateralised_visual_memories

For manuscripts utilizing custom algorithms or software that are central to the research but not yet described in published literature, software must be made available to editors and reviewers. We strongly encourage code deposition in a community repository (e.g. GitHub). See the Nature Portfolio guidelines for submitting code & software for further information.

Data

Policy information about availability of data

All manuscripts must include a data availability statement. This statement should provide the following information, where applicable:

- Accession codes, unique identifiers, or web links for publicly available datasets
- A description of any restrictions on data availability
- For clinical datasets or third party data, please ensure that the statement adheres to our policy

Data used in this study are available on a Github repository https://github.com/antnavteam/lateralised_visual_memories

Research involving human participants, their data, or biological material

Policy information about studies with human participants or human data. See also policy information about sex, gender (identity/presentation), and sexual orientation and race, ethnicity and racism.

Reporting on sex and gender	*Use the terms sex (biological attribute) and gender (shaped by social and cultural circumstances) carefully in order to avoid confusing both terms. Indicate if findings apply to only one sex or gender; describe whether sex and gender were considered in study design; whether sex and/or gender was determined based on self-reporting or assigned and methods used.*<br>*Provide in the source data disaggregated sex and gender data, where this information has been collected, and if consent has been obtained for sharing of individual-level data; provide overall numbers in this Reporting Summary. Please state if this information has not been collected.*<br>*Report sex- and gender-based analyses where performed, justify reasons for lack of sex- and gender-based analysis.*
Reporting on race, ethnicity, or other socially relevant groupings	*Please specify the socially constructed or socially relevant categorization variable(s) used in your manuscript and explain why they were used. Please note that such variables should not be used as proxies for other socially constructed/relevant variables (for example, race or ethnicity should not be used as a proxy for socioeconomic status).*<br>*Provide clear definitions of the relevant terms used, how they were provided (by the participants/respondents, the researchers, or third parties), and the method(s) used to classify people into the different categories (e.g. self-report, census or administrative data, social media data, etc.)*<br>*Please provide details about how you controlled for confounding variables in your analyses.*
Population characteristics	*Describe the covariate-relevant population characteristics of the human research participants (e.g. age, genotypic information, past and current diagnosis and treatment categories). If you filled out the behavioural & social sciences study design questions and have nothing to add here, write "See above."*
Recruitment	*Describe how participants were recruited. Outline any potential self-selection bias or other biases that may be present and how these are likely to impact results.*
Ethics oversight	*Identify the organization(s) that approved the study protocol.*

Note that full information on the approval of the study protocol must also be provided in the manuscript.

Field-specific reporting

Please select the one below that is the best fit for your research. If you are not sure, read the appropriate sections before making your selection.

☐ Life sciences ☐ Behavioural & social sciences ☒ Ecological, evolutionary & environmental sciences

For a reference copy of the document with all sections, see nature.com/documents/nr-reporting-summary-flat.pdf

Ecological, evolutionary & environmental sciences study design

All studies must disclose on these points even when the disclosure is negative.

Study description	The study is composed of 3 behavioural experiments in the field (plus computational models).<br>-Experiment 1 looks at the turning response of homing ants mounted on the trackball when facing different direction relative to their route familiar goal, when placed on their route or on unfamiliar terrain.<br>-Experiment 2 looks at the change of turning response of homing ants mounted on the trackball in a given direction relative to their route (or on unfamiliar terrain) when subjected to a sudden rotation in the sun apparent position (using a mirror).<br>-Experiment 3 look at the change in paths characteristics (mearnder and bearing across time) of ants homing (on the floor) along their familiar route that are subjected to a sudden rotation of the sun direction in the sky.<br>Individual ants were tested once in a single experiment only.
Research sample	The research samples are groups of individuals foragers of Cataglyphis velox and Myremecia croslandi ants. Those ants are solitary foragers and expert visual navigators, thus making them good candidates to study navigation mechanisms.
Sampling strategy	For Experiment 1 and 2, given the straightforwardness of the response analysed (turn left or right) and the use of non parametrical statsitic, we favour a low N (<20) as we tested the existend of a clear-cut mechanism and thus a strong effect. Sampling strategies across group is achieved by sequentially interleaving individual's random attribution to the different group condition. For group condition were we expected no effect, the n was doubled (sampled twice more often) to avoid type II errors, this results in lower n for the group conditions where we expected an effect, thus decreasing the risk of type I errors.<br>For experiment 3 (figure 4), we set a slightly higher Ns (around 25 per condition) given that we explored the change of path characteristics of ant resulting from complex npn-linear dynamics, and thus wihtout clear prior notion of the strengh of the effect. N=25 remains sufficiently low to guaranty a strong effect and thus an ecological relevance if significant.
Data collection	An ant was picked from the foraging area, marked individually using paint dots, and a metallic paint dot was placed on her thorax, the ant was then mounted on the trackball system (attached to a tiny magnet), the platform containing the trackball and the ant was placed on the desired position (either on their familiar route or <100m away from their nest). Recording started, and the bucket surrounding the set up was lifted, revealing the scene to the ant, which was recorded up to 20s before being released free again.<br>Data collection was done by the all the authors, across several field trips.

Timing and spatial scale	Data collection for experiment 1 was achieved in June to July 2018 for C. velox and Feb. to Mar. 2019 for Myrmecia croslandi.<br>Data collection for experiment 2 was achieved in June to July 2018.<br>Data collection for experiment 3 was achieved in June to July 2019.<br>The experiments were carried out all week days during the ant foraging activity (10am till 6pm) and the time necessary to test all our sujects in the desired conditions.
Data exclusions	For experiment 1 and 2 (on trackball). We excluded from the analysis the first 3sec after the bucket surrounding the set up was lifted. The reason was to provide time for the experimenter to leave the scene, and ensure the ant response analysed could not be bias by this potential perturbation.
Reproducibility	The results of the experiment 1 where successfully repeated on two different species (Catalgyphis velox and Myrmecia croslandi), as well as repeated in Experiment 2a (which builds upon experiment 1). The effect observed in experiment 2 (effect of the sun mirroring) where repeated in experiment 3 with ant running on the floor. Thus all attempts to repeat the experiments were successful.
Randomization	For experiment 1,2,3. Ants were randomly selected from the foraging area and attributed to one of the conditions in a random order. For experiment 1, ants were subjected consecutively to 8 different orienatation of the trackball system drawn in a pseudo random order: ensuring that the transition between orientation was random within individual, but that each orientation sequence number was balance across the population.
Blinding	Blinding was not relevant to our study because the data on ants locomotion used in this study were recorded by laser sensors and not a human experimentator.

Did the study involve field work? ☒ Yes ☐ No

Field work, collection and transport

Field conditions	The studies were conducted in two field site, one south of Seville (el Copéro) under semi-arid conditions (temperature 30-38°C), without rainfall. One in Australian Natiaonal University grounds in a woodland area of the campus (25-32°C, no rainfall).
Location	Study on Cataglyphis velox : 37.332760, -5.989039. Study on Myrmecia croslandi : -35.280382, 149.105719
Access & import/export	Both access where achieved through collabortaion with local research facility approval (see aknowledgement).<br>Data were only monitored on cameras and computer, no biological sample were taken.
Disturbance	Our equipements (camera on tripod, laptop and trackball setup) was small and deployed and taken away everyday. All ant tested were released after the behavioural recording, and without being harmed.

Reporting for specific materials, systems and methods

We require information from authors about some types of materials, experimental systems and methods used in many studies. Here, indicate whether each material, system or method listed is relevant to your study. If you are not sure if a list item applies to your research, read the appropriate section before selecting a response.

Materials & experimental systems

n/a	Involved in the study	
☒	☐	Antibodies
☒	☐	Eukaryotic cell lines
☒	☐	Palaeontology and archaeology
☐	☒	Animals and other organisms
☒	☐	Clinical data
☒	☐	Dual use research of concern
☒	☐	Plants

Methods

n/a	Involved in the study	
☒	☐	ChIP-seq
☒	☐	Flow cytometry
☒	☐	MRI-based neuroimaging

Animals and other research organisms

Policy information about studies involving animals; ARRIVE guidelines recommended for reporting animal research, and Sex and Gender in Research

Laboratory animals	The study did not involve laboratory animals
Wild animals	Individuals foragers of Cataglyphis velox ants and Myrmecia croslandi ants were captured as they navigated in their natural environment, received a dot of acrilic paint on their thorax, were given a cookie (to ensure homing motivation), were placed on the trackball setup, for one condition (maximum 2min), released again (upon which they return to their nest with the cookie) and never

	tested again.
Reporting on sex	Because in ants foragers are only females, our study is only using female subjects.
Field-collected samples	NA
Ethics oversight	Because ants are insects no ethical approval or guidance was required for this study. Cataglyphis velox and Myrmecia croslandi are not listed as an endegered species. No individual ants were killed.

Note that full information on the approval of the study protocol must also be provided in the manuscript.

Plants

Seed stocks	*Report on the source of all seed stocks or other plant material used. If applicable, state the seed stock centre and catalogue number. If plant specimens were collected from the field, describe the collection location, date and sampling procedures.*
Novel plant genotypes	*Describe the methods by which all novel plant genotypes were produced. This includes those generated by transgenic approaches, gene editing, chemical/radiation-based mutagenesis and hybridization. For transgenic lines, describe the transformation method, the number of independent lines analyzed and the generation upon which experiments were performed. For gene-edited lines, describe the editor used, the endogenous sequence targeted for editing, the targeting guide RNA sequence (if applicable) and how the editor was applied.*
Authentication	*Describe any authentication procedures for each seed stock used or novel genotype generated. Describe any experiments used to assess the effect of a mutation and, where applicable, how potential secondary effects (e.g. second site T-DNA insertions, mosiacism, off-target gene editing) were examined.*
